# Markers of bone turnover are elevated in patients on antiretrovirals independent of the substance used

**DOI:** 10.1186/1758-2652-13-S4-P97

**Published:** 2010-11-08

**Authors:** RJ Piso, M Rothen, JP Rothen, M Stahl

**Affiliations:** 1 Medizinische Klinik, Kantonsspital Olten, Baslerstrasse 150, Olten, Switzerland; 2 Medical Laboratories, Olten, Switzerland; 3 Kantonsspital Olten, Department of Medicine, Olten, Switzerland

## Objectives

Osteoporosis and bone fractures are correlated to antiretroviral treatment. It is not clear, if some substances inherit greater risk of bone loss than others.

## Methods

We measured pyridinoline, desoxipyridinoline crosslinks and bone specific alkaline phosphatase in 108 HIV positive patients. We compared patients with and without antiretroviral treatment. We then analysed patients with vs. without tenofovir and patients with PI vs NNRTI use.

## Results

Bone specific alk. phosphatase, pyridonoline and desoxipiridinoline crosslinks and were significantly higher in patients with ART compared with patients without ART: 25.17 vs 13.22 pg/L (p < 0.001); 83.64 vs 51.23 nmol/mmol (p < 0.001) and 16.38 vs 9.68 nmol/mmol (p<0.001)respectively. In contrast, no difference was found in patients with vs. without tenofovir 26.25 vs 20.18 pg/l (p =0.08); 80.74 vs 85.83 nmol/mmol (p=0.42) and 19.36 vs 14.00 nmol/mmol (p=0.13) respectively. Comparison between patients with proteinase inhibitor vs non-nucleoside inhibitor yielded no difference either 23.07 vs 26.49pg/l (p=0.33); 94.31 vs 80.65 nmol/mmol (p=0.34) and 18.18 vs 16.29 nmol/mmol (p= 0.52).

## Conclusions

Markers for bone turn over are higher in treated vs untreated patients. No difference concerning tenofovir use or proteinase inhibitor vs. non-nucleoside inhibitor use could be found.

